# Supplementary material for: The Isolation, Identification and Immobilization Method of Three Novel Enzymes with Diosgenin-Producing Activity Derived from an Aspergillus flavus
Source: Int J Mol Sci. 2023 Dec 18;24(24):17611. doi: 10.3390/ijms242417611 (PMC10743735; doi:10.3390/ijms242417611)
Supplement: Supplementary file 1 [file ijms-24-17611-s001.zip › ijms-2714420-supplementary.pdf]

Supplementary Materials

Table S1 Separation result of dioscin-glycosidases (DGAs) by ammonium sulfate with different concentration.

**Ammonium sulfate concentration**	**Total protein (mg)**	**Total vitality (U/mL)**	**Specific activity (U/mg)**
10%	7.6	3.3	4.3
20%	9.78	5.1	5.2
30%	11.45	6.98	6.1
35%	16.89	13.85	8.2
40%	18.11	15.93	8.8
45%	19.35	18.77	9.7
50%	24.7	29.9	12.1
55%	26.2	33.53	12.8
60%	28.3	37.92	13.4
65%	30.46	48.74	16
70%	31.57	46.09	14.6
75%	32.36	36.89	11.4
80%	33.05	30.41	9.2
85%	33.96	23.43	6.9
90%	34.1	19.78	5.8

Table S2 Separation result of DGAs with the number of salting-out

**Number of salting-out**	**Total protein (mg)**	**Total vitality (U/mL)**	**Specific activity (U/mg)**
Crude enzyme liquor	37.43	56.145	15
1	30.46	48.74	16
2	22.44	38.5	17.2
3	13.8	24.56	17.8

Note: Sample 1: enzyme solution precipitated once by ammonium sulfate; Sample 2: enzyme solution precipitated twice by ammonium sulfate; Sample 3: enzyme solution precipitated 3 times by ammonium sulfate.

Table S3 Separation result of DGAs with different salting-out methods

**Salting-out method**	**Total protein (mg)**	**Total vitality (U/mL)**	**Specific activity (U/mg)**
Crude enzyme liquor	37.43	56.145	15

One-step salting-out	30.46	48.74	16
Gradient salting-out	18.89	48.50	25.72

S4 Molecular weight determination of DGAs

The secondary recovered enzyme solution was subjected to SDS-PAGE, staining and decolorization. The migration distances of bromophenol blue indicator and standard protein were measured, the logarithm of the molecular weight of the standard protein (lg MW) was calculated, and the relative mobility (mR) of the standard protein was calculated according to the formula, and the protein molecular weight standard curve was plotted with the relative mobility as the horizontal coordinate and the logarithm of the protein molecular weight as the vertical coordinate. Measure the migration distance of the target protein and calculate the molecular weight of the target enzyme according to the protein molecular weight standard curve.

$$\text{relative mobility} = \frac{\text{Protein sample migration distance (cm)}}{\text{Bromophenol blue migration distance (cm)}} \times 100\% \quad (1)$$

S5

Query length：1187aa

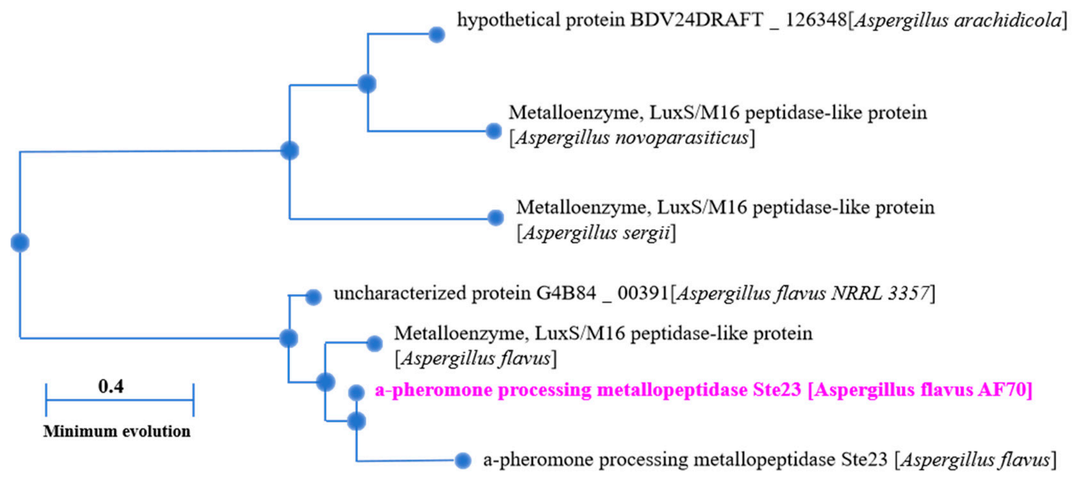

Query length： 910aa

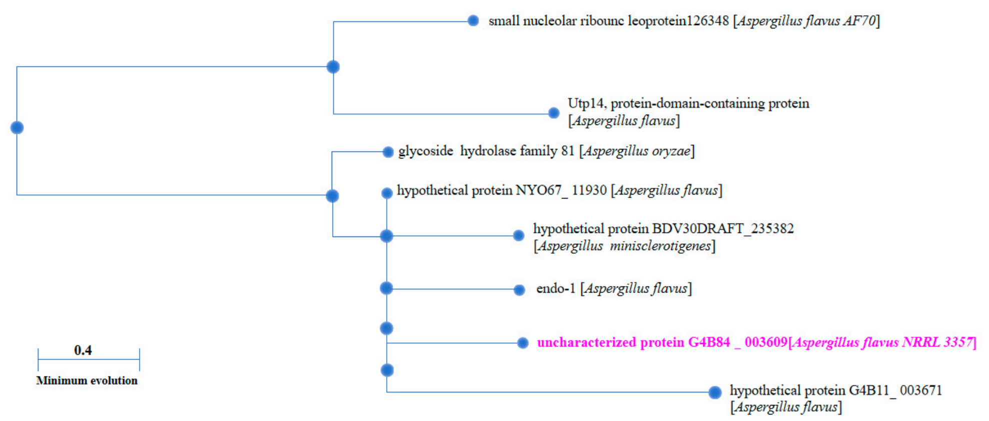

Query length： 476aa

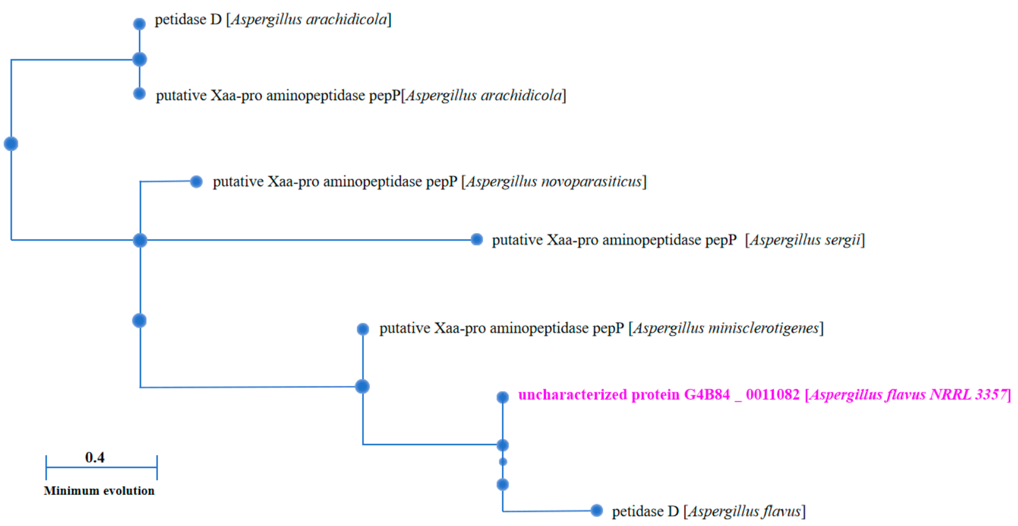

Figure. S1 The alignments and distance tree of the three proteins with their closest homologous proteins.

S6 Optimization of immobilization conditions single-factor experiment

(i) The effect of sodium alginate concentration on the activity of immobilized enzymes at various concentrations ranging from 2.0%, 2.5%, 3.0%, 3.5% and 4.0% .

The immobilized enzyme and the same amount of free enzyme were respectively reacted with saponin solution at 50°C and pH 7.0 for 12 h, and then the reaction was terminated by boiling water bath. The content of diosgenin in samples and the activity

of immobilized and free enzymes were calculated. Each group of experiments was parallel three times, and the average of the results was taken. The effect of sodium alginate concentration on immobilized enzyme activity was evaluated with the recovery rate of enzyme activity (%) as the index.

(ⅱ) The immobilized enzyme activities of DGAs were measured by altering the concentration of $CaCl_2$ solution to 2.0%, 2.5%, 3.0%, 3.5%, and 4.0%. The effect of $CaCl_2$ concentration on the immobilized enzyme activity was evaluated using the enzyme activity recovery (%) as an index.

(ⅲ) Mixed 6mL of 3% sodium alginate solution with 2mL of DGAs solution (pH7.0). The mixture was injected into the syringe at a place about 5 cm above the 3.0% $CaCl_2$ solution, and cured in a 4 °C refrigerator for 0.5 h, 1.0 h, 1.5 h, 2.0 h, 2.5 h, and 3.0 h, respectively. After curing, the microspheres were washed with deionized water for future use. The immobilized enzyme and the same amount of free enzyme were respectively reacted with saponin solution at 50°C and pH 7.0 for 12 h, then the reaction was terminated by boiling water bath. The content of diosgenin in samples and the activity of immobilized and free enzymes were calculated. The effect of immobilization time on immobilized enzyme activity was evaluated with recovery rate (%) as index.

(ⅳ) DGAs were extracted from CAC-NAAC buffer at pH 6.0, 7.0, 8.0, 9.0 and 10.0, and 0.02 M. A total of 6 mL of 3% sodium alginate solution was mixed with 2 mL of DGAs at different pH. The mixture was injected into the syringe at a place about 5 cm above the 3.0% $CaCl_2$ solution, and cured in a 4°C refrigerator for 1 h. Then the microspheres were washed with deionized water for future use. The immobilized enzyme and the same amount of free enzyme were respectively reacted with saponin solution at 50°C and pH 7.0 for 12 h, and then the reaction was terminated by boiling water bath. The content of diosgenin in samples and the activity of immobilized and free enzymes were calculated. Each group of experiments was parallel three times, and the average of the results was taken. The effect of pH on the activity of immobilized enzyme was evaluated using the recovery rate of enzyme activity (%) as index.

Results

It can be seen from Fig. S7A that the activity of the immobilized enzyme increased and then decreased with the increase in sodium alginate concentration, and

the activity of enzyme was at a maximum when the concentration of sodium alginate was 3%. This may be due to the fact that at a concentration of sodium alginate of 3% or less, the immobilized microsphere surface formation of the membrane strength is not strong enough, the mechanical strength is poor, the microsphere is fragile and it is easy to break through its wall, resulting in the loss of the enzyme during the washing, resulting in low enzyme activity; a sodium alginate concentration of 3% or more, the viscosity of the sodium alginate solution is too large, the surface of the microsphere prepared by the structure is tight, the enzyme cannot be in contact with the saponin substrate, resulting in the decline in DGAs activity. Therefore, 3% sodium alginate was chosen for the subsequent test.

It can be observed that the enzyme activity of the immobilized DGAs increased and then decreased with the increase in $CaCl_2$ concentration, and the enzyme activity was at a maximum when the $CaCl_2$ concentration was 3.0 %, as shown in Fig. S7B. This may be because when the $CaCl_2$ concentration is lower than 3%, the mechanical strength of the immobilized gel microspheres is relatively poor, and the microspheres are fragile and it is easy to break their walls, which causes the loss of enzymes during the washing, resulting in lower enzymatic activity; when the $CaCl_2$ concentration is greater than 3%, the surface of the gel microspheres is covered with Ca^{2+}, which mi tigates the impact of the enzymes and leads to a decrease in the recovery of enzyme activity. Therefore, 3% $CaCl_2$ was chosen for the subsequent test.

It can be seen from Fig. S7C that within a certain range, the activity of immobilized DGAs increased and then decreased with the increase in immobilization time, and the enzyme activity was maximum when immobilized for 1 h. The enzyme activity of immobilized DGAs increased with the increase in immobilization time and then decreased. This may be due to the fact that when the fixation time was lower than 1 h, the fixation time was too short, the embedding process was incomplete, and some enzymes were eluted during washing without being immobilized, which resulted in a lower recovery of enzyme activity; when the fixation time was higher than 1 h, the immobilized microspheres were compact, and the contact area between the enzyme and the saponin substrate was reduced, which led to a decrease in the recovery of enzyme activity. Therefore, a fixation time of 1 h was chosen for the next operation.

Within a certain range, the activity of the immobilized DGAs increased and then decreased with the increase in pH, and the enzyme activity was at a maximum at an immobilized pH of 8 as shown in Fig. S7D. This may be due to the ionization of the characteristic groups on the surface of the enzyme at a different pH, which makes the enzyme molecules exist in different dissociated states and affects the reaction between the enzyme and the saponin substrate therefore, the immobilized pH of 8 was chosen for the next step in this experiment.

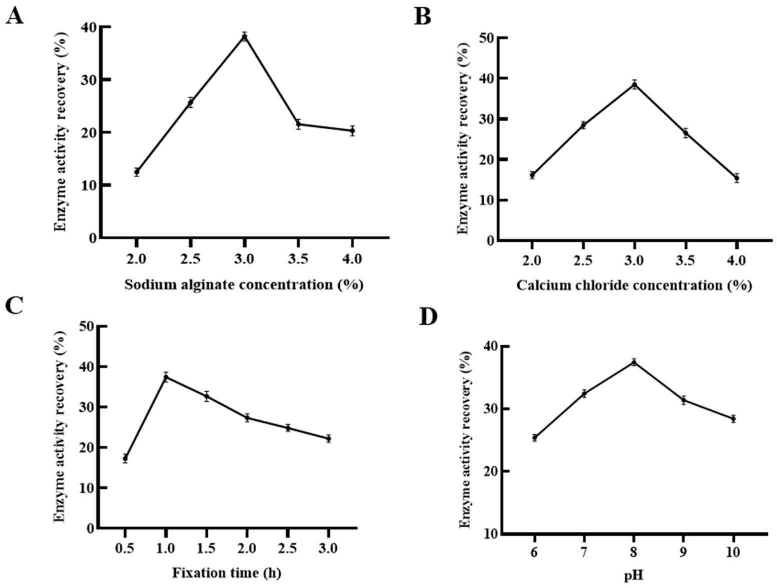

Figure S2 Optimization of immobilization conditions

A**:** Effect of sodium alginate concentration on immobilization of DGAs. B: Effect of $CaCl_2$ concentration on immobilization of DGAs. C: Effect of immobilization time on immobilization of DGAs. D: The effect of pH on the immobilization of DGAs.
